# Supplementary material for: Integrating genomic structural equation modeling and experimental validation to unravel the genetic basis of male genital lichen sclerosus
Source: Front Immunol. 2026 Jul 16;17:1857945. doi: 10.3389/fimmu.2026.1857945 (PMC13435250; doi:10.3389/fimmu.2026.1857945)
Supplement: Supplementary file 6 [file SupplementaryFile5.zip › result/fuma_PSYCH_factor_sCCA3_focus.chr2.chr2.8422147.10298469.pop1.pdf]

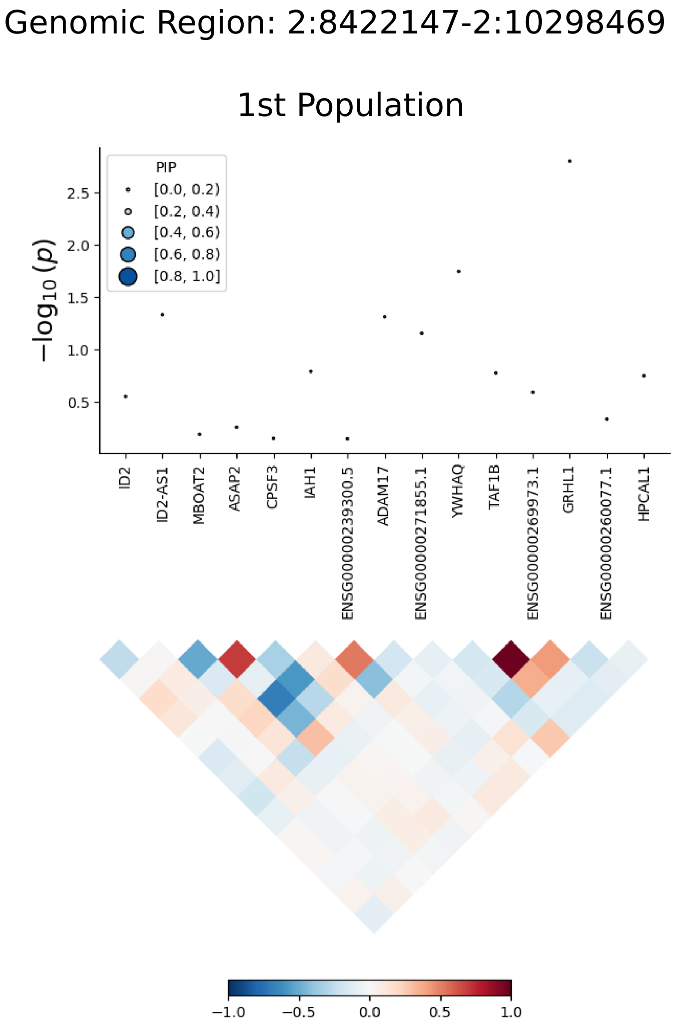
Genomic Region: 2:8422147-2:10298469
1st Population
PIP
[0.0, 0.2)
[0.2, 0.4)
[0.4, 0.6)
[0.6, 0.8)
[0.8, 1.0]
$-\log_{10}(p)$
2.5
2.0
1.5
1.0
0.5
ID2
ID2-AS1
MBOAT2
ASAP2
CPSF3
IAH1
ENSG00000239300.5
ADAM17
ENSG00000271855.1
YWHAQ
TAF1B
ENSG00000269973.1
GRHL1
ENSG00000260077.1
HPCAL1
−1.0
−0.5
0.0
0.5
1.0
